# Clinical and Dermoscopic Evaluation of Patients With Disorders of Hypopigmentation and Depigmentation

**DOI:** 10.7759/cureus.117133

**Published:** 2026-09-29

**Authors:** Aayush Goel, Aneet Mahendra, Sanjeev Gupta, Hritik V Shah, Nitish Goyal

**Affiliations:** 1 Dermatology, Maharishi Markandeshwar Institute of Medical Sciences and Research, MMDU Mullana, Ambala, IND

**Keywords:** dermoscopy, hypopigmentary disorders, nevus depigmentosus, pityriasis alba, pityriasis versicolor, vitiligo, wood’s lamp

## Abstract

Background: Hypopigmentary and depigmentary disorders frequently present with overlapping clinical features, often making diagnosis challenging. Dermoscopy serves as a valuable non-invasive tool that provides additional morphological information by revealing subsurface morphological patterns.

Objectives: The aim of this study was to evaluate the clinical and dermoscopic features of various hypopigmentary and depigmentary disorders encountered in routine clinical practice.

Material and methods: A hospital-based observational cross-sectional study was conducted in 200 patients (aged 6-70 years, both genders) presenting with hypopigmentary and depigmentary disorders in a tertiary care dermatology outpatient department. All patients underwent detailed clinical examination, Wood’s lamp examination, 10% potassium hydroxide (KOH) examination where indicated, and dermoscopic evaluation. Histopathological examination was performed in doubtful cases.

Results: Among 200 patients, the majority belonged to the 11-20-year age group (33.5%). Vitiligo (36.5%), pityriasis versicolor (35.0%), and pityriasis alba (15.0%) were the most common disorders observed, whereas idiopathic guttate hypomelanosis, nevus depigmentosus, contact leukoderma, and leprosy were observed in a smaller number of patients. On Wood’s lamp examination, vitiligo demonstrated bluish-white fluorescence, while pityriasis versicolor showed greenish-yellow to yellow fluorescence. KOH examination was positive in 81.4% of pityriasis versicolor cases with “spaghetti and meatballs” appearance. Vitiligo demonstrated absent pigment network (69.6%) and diffuse white glow (67.1%) on dermoscopy. Idiopathic guttate hypomelanosis showed margin patterns like amoeboid, feathery, and petaloid margins. Pityriasis versicolor showed brownish-white background (94.3%), faint pigment network (80.0%), and scaling accentuated in skin creases (72.9%). Pityriasis alba demonstrated ill-defined margins (93.3%) and a faint pigment network (73.3%). Leprosy lesions revealed yellowish-orange structureless areas (80.0%) with a distorted pigment network (60.0%).

Conclusion: Dermoscopy is a valuable adjunctive, non-invasive tool in differentiating clinically similar hypopigmentary and depigmentary disorders. Combined with Wood’s lamp examination and KOH microscopy, it significantly improves diagnostic precision and may reduce the need for invasive investigations.

## Introduction

Hypopigmented and depigmented disorders comprise a heterogeneous group of acquired and congenital conditions characterized by partial or complete loss of skin pigmentation [1]. These disorders are frequently encountered in dermatological practice and often pose a diagnostic challenge because of their overlapping clinical presentations despite distinct underlying etiologies. Dermoscopy has emerged as a valuable non-invasive diagnostic technique that enables in vivo visualization of subsurface skin structures not visible to the naked eye [2]. The present study was undertaken to evaluate dermoscopic patterns in various hypopigmentary and depigmentary disorders encountered in routine clinical practice.

## Materials and methods

Study design

An observational, cross-sectional study design was adopted.

Study area

The study included patients with hypopigmented and depigmented lesions. These patients presented to the Dermatology Outpatient Clinic at Maharishi Markandeshwar Institute of Medical Sciences and Research (MMIMSR), Mullana, Ambala, Haryana, India.

Study population and sample size

A total of 200 consecutive patients of either gender, aged between 6 and 70 years, with hypopigmentary and depigmentary disorders attending the dermatology outpatient department were included.

Ethical considerations

Institutional Ethics Committee approval was obtained before the commencement of the study (IEC-3224, dated February 18, 2025). Patients were enrolled after receiving an in-depth explanation about the nature of the study, and informed consent was obtained, reassuring confidentiality of the information shared.

Study measures

Data collected included demographic details, detailed history, clinical examination, and Wood’s lamp examination. Potassium hydroxide examination was performed in scaly lesions, while histopathological examination was reserved for diagnostically doubtful cases. Dermoscopic examination was carried out in all patients using a polarized dermoscope, Dermoscope AM7515MZT (Dino-Lite Edge) with variable magnification. Features evaluated included background color, pigment network alterations, scaling patterns, lesion margins, follicular findings, perifollicular changes, and vascular structures. Dermoscopic terminology was standardized according to previously published descriptions [2-4]. A faint pigment network was defined as a uniformly pale but preserved reticular pattern, whereas a reduced pigment network denoted attenuation of the network with decreased prominence but preserved continuity [3,5,6]. A reversed pigment network was characterized by depigmented reticular lines surrounding relatively preserved pigmentation [3]. Patchy white scaling refers to multiple discrete areas of white scaling distributed over the lesion, whereas focal white scaling refers to localized scaling confined to a small, isolated area within the lesion. Lesion margins were categorized as well-defined, ill-defined, or intermediate according to the clarity of demarcation from the surrounding skin [3,4].

Statistical analysis

Data collected were entered and analyzed using Microsoft Excel (Microsoft Corp., Redmond, WA, USA) and IBM SPSS Statistics version 26 (IBM Corp., Armonk, NY). Descriptive statistics were used to summarize the data. Continuous variables were expressed as mean ± standard deviation, while categorical variables were presented as frequencies and percentages.

## Results

A total of 200 patients with hypopigmentary and depigmentary disorders were included in the study. The mean age was 25.91 ± 15.35 years, with a slight male predominance (103 (51.5%) males and 97 (48.5%) females). Vitiligo was the most common diagnosis, accounting for 73 (36.5%) cases, followed by pityriasis versicolor in 70 (35.0%) patients. Other diagnoses included pityriasis alba, idiopathic guttate hypomelanosis, nevus depigmentosus, contact leukoderma, and leprosy. The distribution of patients according to diagnosis is summarized in Table 1.

**Table 1:** Distribution of patients according to clinical diagnosis (n=200)

Clinical diagnosis	No. of patients	%
Contact leucoderma	6	3.00%
Idiopathic guttate hypomelanosis	9	4.50%
Leprosy	5	2.50%
Nevus depigmentosus	7	3.50%
Pityriasis alba	30	15.00%
Pityriasis versicolor	70	35.00%
Vitiligo	73	36.50%
Total	200	100%

On 10% KOH microscopy, pityriasis versicolor demonstrated the characteristic "spaghetti and meatball" appearance in 57 (81.4%) cases, whereas all cases of pityriasis alba showed negative KOH findings. Wood’s lamp examination demonstrated characteristic fluorescence patterns across the different disorders. The detailed distribution of fluorescence patterns is presented in Table 2.

**Table 2:** Distribution of patients according to fluorescence pattern on Wood’s lamp (n=200)

Diagnosis	Bluish-white fluorescence	Greenish-yellow to yellow fluorescence	Mild accentuation/minimal fluorescence	No fluorescence
Contact leucoderma (n=6)	3 (50.0%)	0	3 (50.0%)	0
Idiopathic guttate hypomelanosis (n=9)	0	0	9 (100%)	0
Leprosy (n=5)	0	0	2 (40.0%)	3 (60.0%)
Nevus depigmentosus (n=7)	0	0	5 (71.4%)	2 (28.6%)
Pityriasis alba (n=30)	0	0	17 (56.7%)	13 (43.3%)
Pityriasis versicolor (n=70)	0	69 (98.6%)	0	1 (1.4%)
Vitiligo (n=73)	73 (100%)	0	0	0
Total (n=200)	76 (38.0%)	69 (34.5%)	36 (18.0%)	19 (9.5%)

Dermoscopic examination revealed a diffuse white glow with an absent pigment network in vitiligo, scaling accentuated within skin creases in 51 (72.9%) cases of pityriasis versicolor, preservation of the reticular pigment network in nevus depigmentosus, and yellowish-orange structureless areas in leprosy. Idiopathic guttate hypomelanosis showed margin patterns such as amoeboid, feathery, and petaloid in 4 (44.4%), 3 (33.3%), and 2 (22.2%) cases, respectively. Representative clinical and dermoscopic findings are illustrated in Figures 1-5, while the complete dermoscopic characteristics are summarized in Table 3.

**Figure 1:**
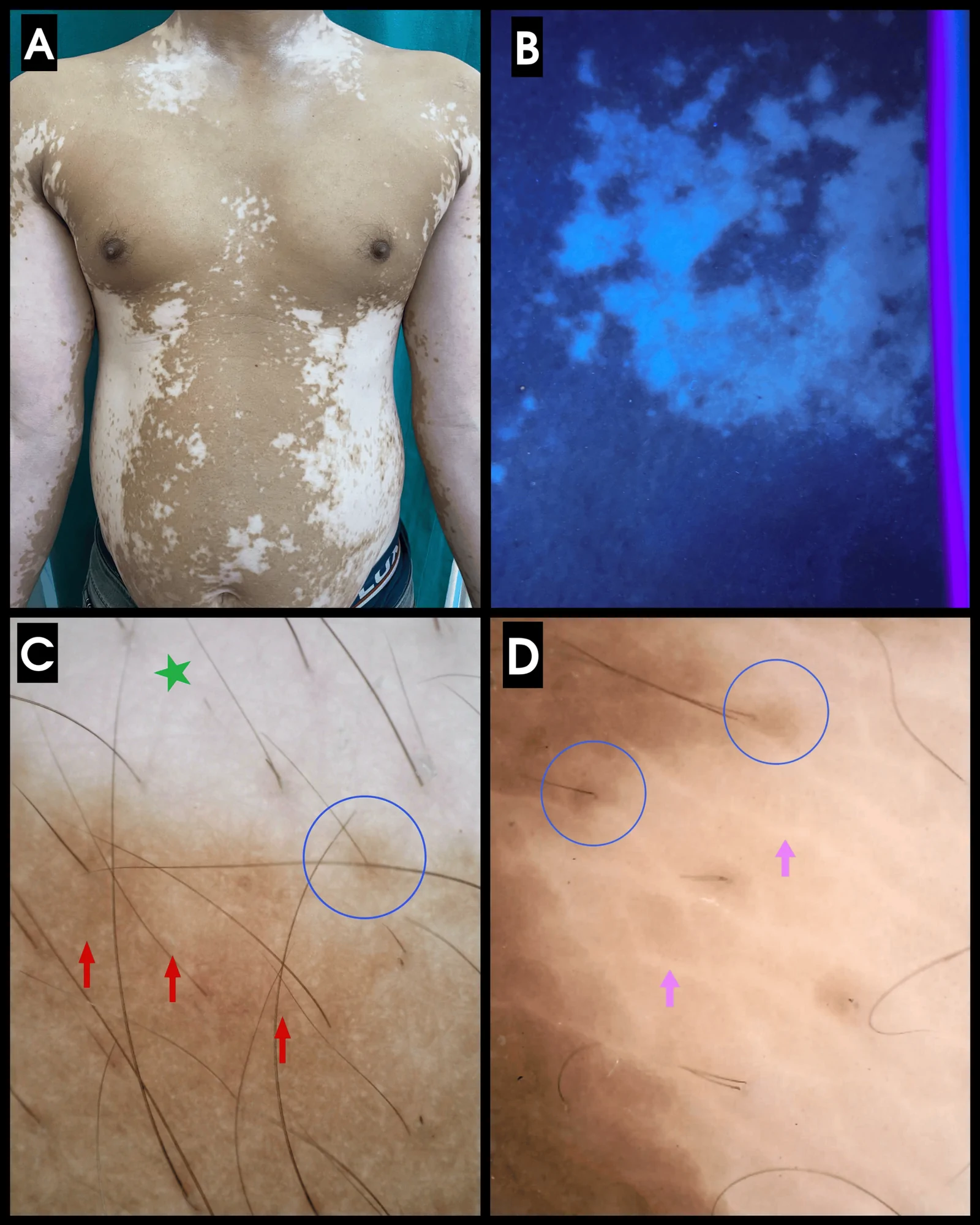
Clinical, Wood’s lamp, and dermoscopic pictures of vitiligo (A) Clinical image showing depigmented patches of vitiligo vulgaris. (B) Wood’s lamp image showing a depigmented vitiligo patch with bright blue-white accentuation under ultraviolet illumination. (C, D) Dermoscopic images (10× magnification) of vitiligo lesions showing diffuse white glow with complete loss of pigment network (green star), reversed pigment network (purple arrows), ill-defined margins (red arrows), and peri-follicular pigmentation (blue circles).

**Figure 2:**
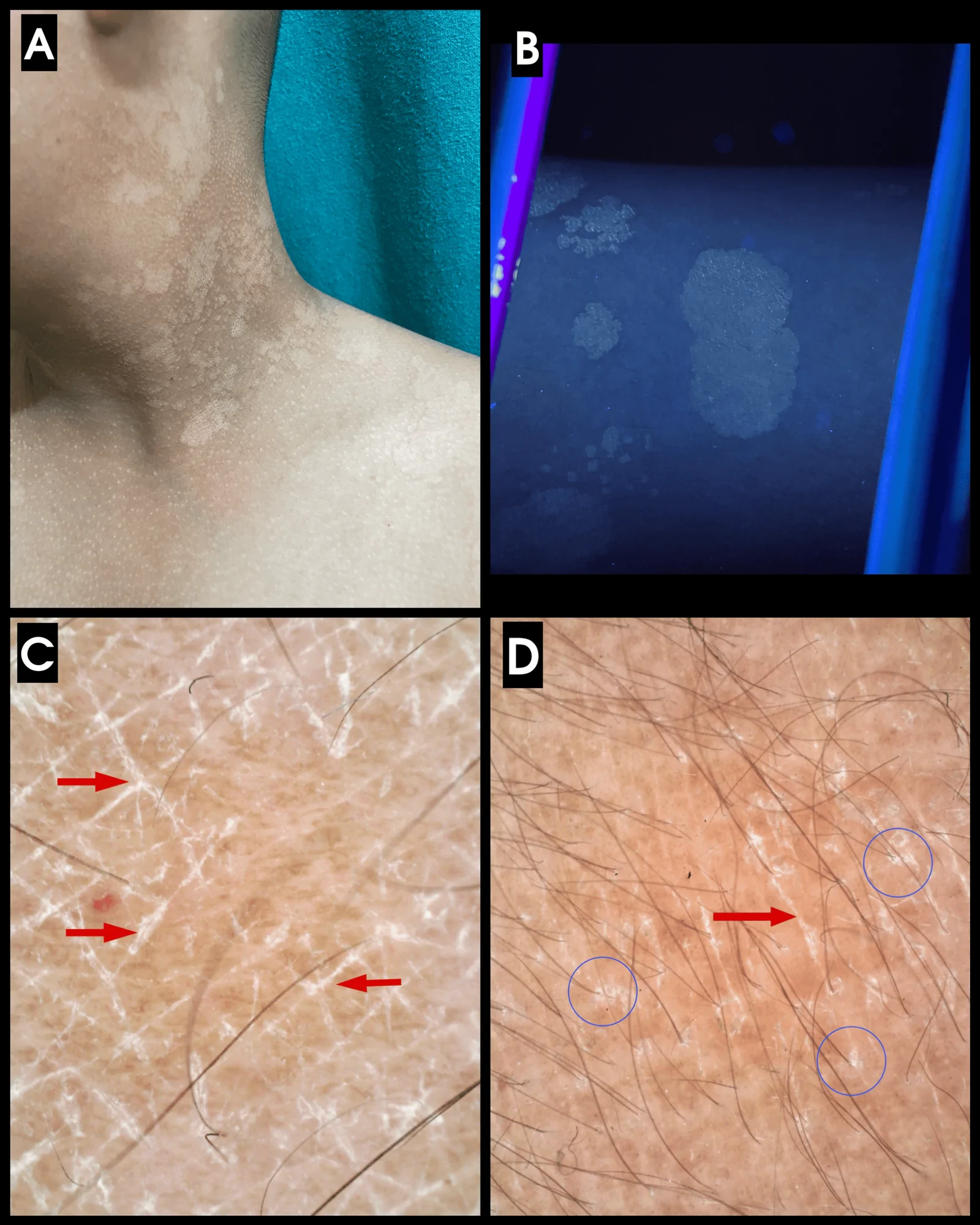
Clinical, Wood’s lamp, and dermoscopic pictures of pityriasis versicolor (A) Clinical image showing pityriasis versicolor over the neck area. (B) Wood’s lamp examination showing a greenish-yellow fluorescence under ultraviolet illumination. (C, D) Dermoscopic images (10× magnification) of pityriasis versicolor showing a brownish-white background with a faint pigment network, accentuated scaling at skin creases (red arrows), and peri-follicular scaling (blue circles)

**Figure 3:**
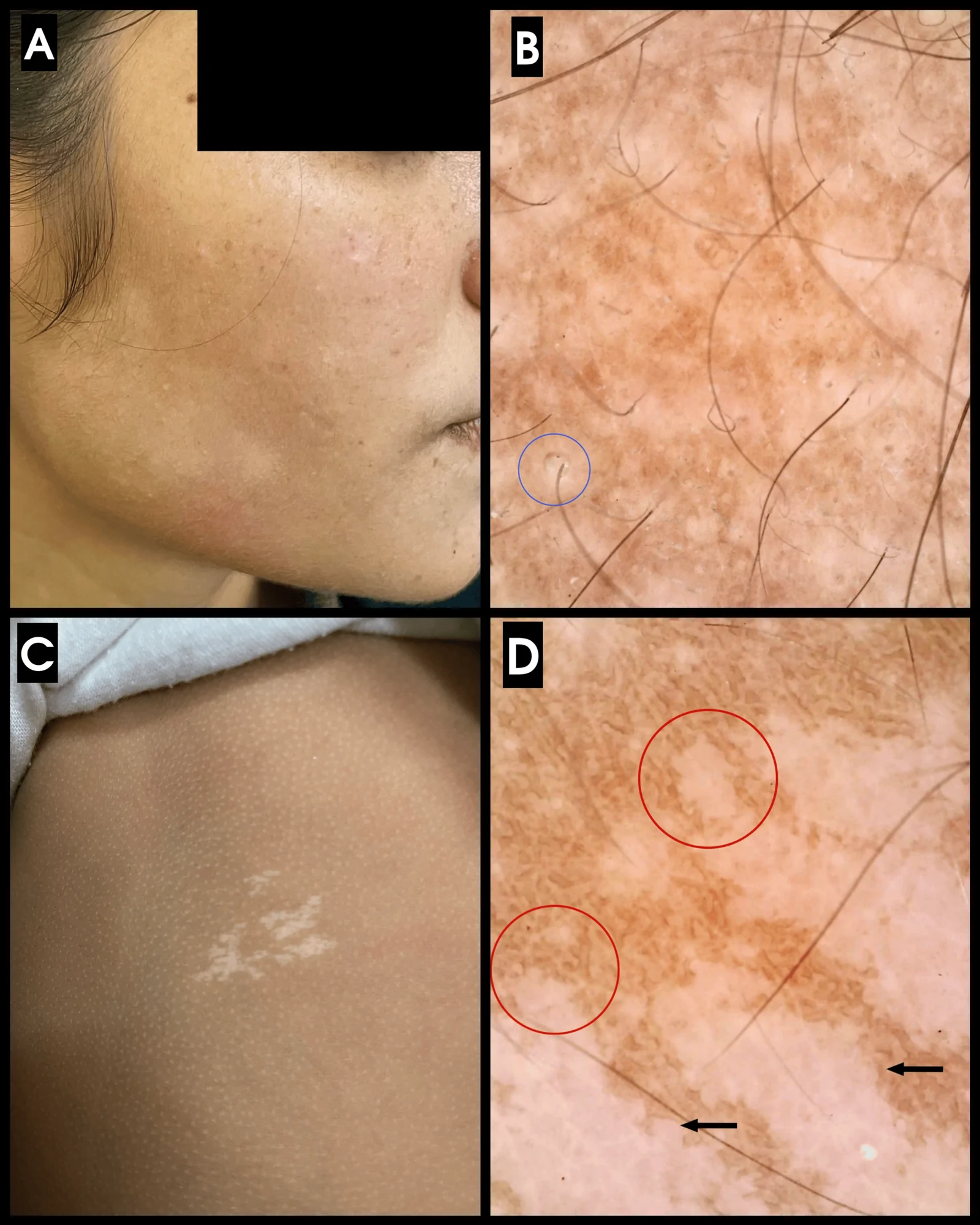
Clinical and dermoscopic pictures of pityriasis alba and nevus depigmentosus (A) Clinical images of pityriasis alba showing multiple hypopigmented patches on the face. (B) Dermoscopic image (10× magnification) of pityriasis alba showing a brownish-white background with a faint pigment network and perifollicular scaling (blue circle). (C) Clinical image of nevus depigmentosus on back. (D) Dermoscopic image (10× magnification) of nevus depigmentosus showing feathery margins (black arrows) with pseudopod-like extensions (red circles).

**Figure 4:**
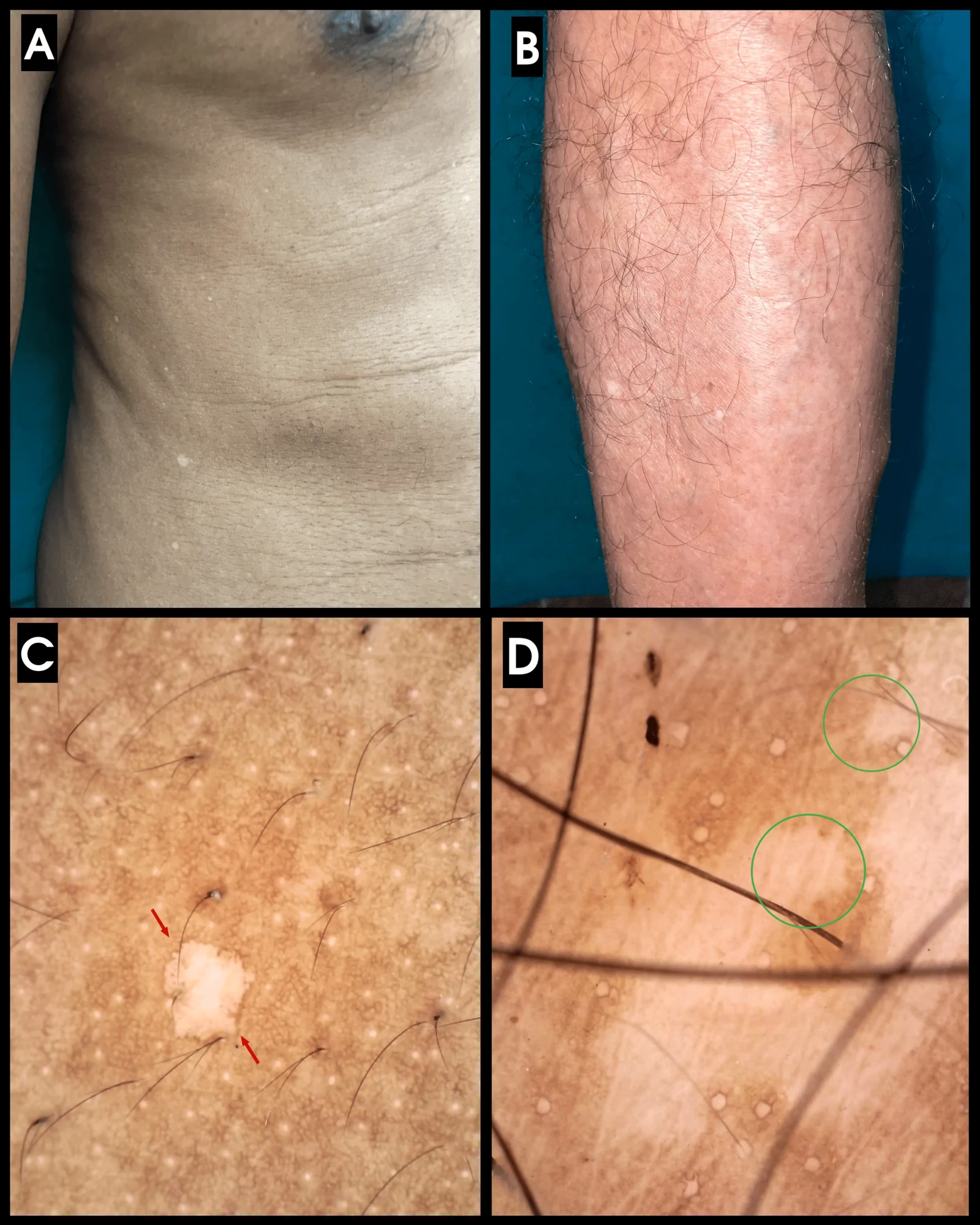
Clinical and dermoscopic pictures of idiopathic guttate hypomelanosis (A, B) Clinical images of idiopathic guttate hypomelanosis showing multiple hypopigmented macules over the trunk and leg. (C) Dermoscopic image (10× magnification) showing diffuse white glow with petaloid margins (red arrows). (D) Dermoscopic image (10× magnification) showing amoeboid margins (green circles).

**Figure 5:**
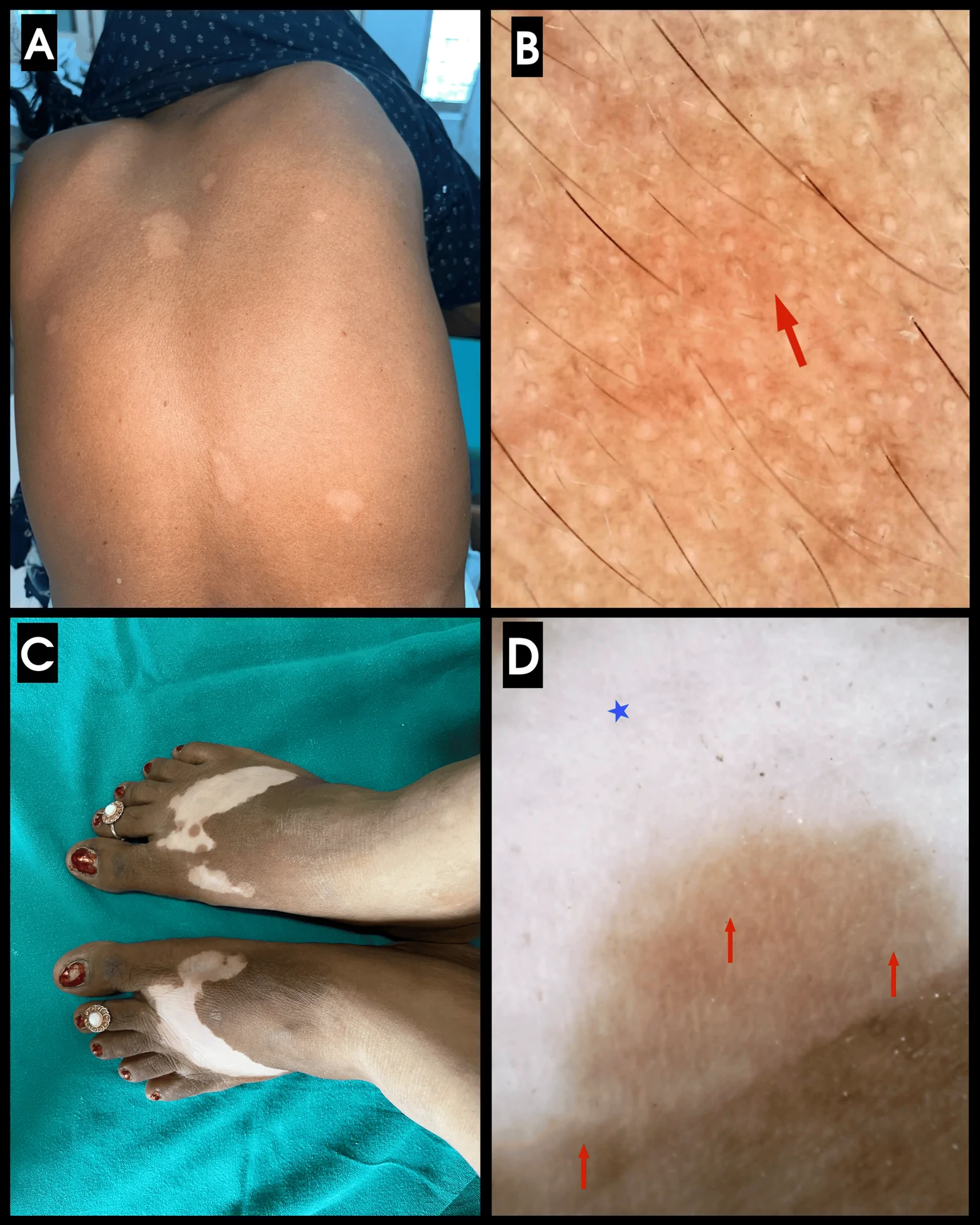
Clinical and dermoscopic pictures of leprosy and contact leucoderma (A, B) Clinical image and dermoscopic image (10× magnification) of leprosy showing a distorted pigment network and a yellowish-orange structureless area (red arrow). (C, D) Clinical image and dermoscopic image (10× magnification) of contact leukoderma showing a diffuse white glow with an absent pigment network (blue star) and ill-defined margins (red arrows).

**Table 3:** Dermoscopic findings of patients according to clinical diagnosis (n=200) IGH, Idiopathic guttate hypomelanosis.

Dermoscopic finding	Contact leukoderma (n=6)	IGH (n=9)	Leprosy (n=5)	Nevus depigmentosus (n=7)	Pityriasis alba (n=30)	Pityriasis versicolor (n=70)	Vitiligo (n=73)
BACKGROUND COLOR							
Brownish white	0 (0%)	0 (0%)	5 (100%)	6 (85.7%)	28 (93.3%)	66 (94.3%)	0 (0%)
Diffuse white glow	4 (66.7%)	6 (66.7%)	0 (0%)	0 (0%)	0 (0%)	0 (0%)	49 (67.1%)
White	2 (33.3%)	3 (33.3%)	0 (0%)	1 (14.3%)	2 (6.7%)	4 (5.7%)	24 (32.9%)
PIGMENT NETWORK							
Absent	4 (66.7%)	8 (88.9%)	0 (0%)	0 (0%)	0 (0%)	0 (0%)	51 (69.6%)
Reversed	0 (0%)	0 (0%)	0 (0%)	0 (0%)	0 (0%)	0 (0%)	12 (16.4%)
Reduced	2 (33.3%)	1 (11.1%)	2 (40%)	0 (0%)	5 (16.7%)	6 (8.6%)	9 (12.3%)
Faint	0 (0%)	0 (0%)	0 (0%)	3 (42.9%)	22 (73.3%)	56 (80%)	0 (0%)
Distorted	0 (0%)	0 (0%)	3 (60%)	0 (0%)	3 (10%)	8 (11.4%)	1 (1.4%)
Reticular	0 (0%)	0 (0%)	0 (0%)	4 (57.1%)	0 (0%)	0 (0%)	0 (0%)
MARGIN							
Ill defined	5 (83.3%)	0 (0%)	5 (100%)	5 (71.4%)	28 (93.3%)	61 (87.1%)	34 (46.6%)
Intermediate	0 (0%)	1 (11.1%)	0 (0%)	2 (28.6%)	2 (6.7%)	4 (5.7%)	13 (17.8%)
Well defined	1 (16.7%)	8 (88.9%)	0 (0%)	0 (0%)	0 (0%)	5 (7.1%)	26 (35.6%)
SCALING							
Diffuse white scaling	0 (0%)	0 (0%)	0 (0%)	0 (0%)	15 (50%)	37 (52.9%)	0 (0%)
Patchy white scaling	0 (0%)	0 (0%)	0 (0%)	0 (0%)	8 (26.7%)	24 (34.3%)	1 (1.4%)
Focal white scaling	0 (0%)	0 (0%)	0 (0%)	0 (0%)	3 (10%)	5 (7.1%)	2 (2.7%)
Perifollicular scaling	0 (0.0%)	0 (0.0%)	0 (0.0%)	0 (0.0%)	6 (20.0%)	25 (35.7%)	0 (0.0%)
VASCULAR STRUCTURES							
Present	4 (66.7%)	0 (0%)	2 (40%)	0 (0%)	11 (36.7%)	15 (21.4%)	35 (47.9%)
Absent	2 (33.3%)	9 (100%)	3 (60%)	7 (100%)	19 (63.3%)	55 (78.6%)	38 (52.1%)
VESSEL MORPHOLOGY							
Dotted vessels	1 (16.7%)	0 (0%)	0 (0%)	0 (0%)	7 (23.3%)	8 (11.4%)	10 (13.7%)
Linear vessels	3 (50%)	0 (0%)	2 (40%)	0 (0%)	4 (13.3%)	7 (10%)	25 (34.2%)
FOLLICULAR/PERILESIONAL CHANGES							
Leucotrichia	2 (33.3%)	0 (0%)	1 (20%)	0 (0%)	4 (13.3%)	2 (2.9%)	30 (41.1%)
Perifollicular pigmentation	1 (16.7%)	5 (55.6%)	0 (0%)	5 (71.4%)	0 (0%)	2 (2.9%)	30 (41.1%)
Perilesional pigmentation	1 (16.7%)	0 (0%)	0 (0%)	2 (28.6%)	0 (0%)	0 (0%)	22 (30.1%)

## Discussion

Hypopigmentary and depigmentary disorders are among the most common pigmentary conditions encountered in dermatology outpatient practice and often pose a diagnostic challenge because of their overlapping clinical morphology. This challenge is particularly relevant in population in the Indian subcontinent with predominantly darker skin (Fitzpatrick skin phototypes IV and V). Darker skin contains larger, heavily melanized melanosomes with higher epidermal melanin content, conferring greater photoprotection against ultraviolet-induced damage [7]. Although hyperpigmentary disorders are common, hypopigmented lesions are often more alarming because even minimal pigment loss produces a striking contrast with the surrounding skin.

Beyond the cosmetic concern, the sudden appearance of hypopigmented lesions often causes considerable psychological distress because of the fear of conditions such as vitiligo and leprosy, which have been associated with social stigma, discrimination, misconceptions regarding their contagious nature, and adverse effects on interpersonal relationships and marriage prospects [8]. Accurate differentiation of hypopigmentary disorders is therefore essential not only for appropriate management but also for patient reassurance, counseling, and dispelling these misconceptions.

In a busy outpatient setting, dermatologists rely mainly on clinical examination, often supplemented by a hand lens. However, several hypopigmentary and depigmentary disorders share similar morphology, distribution, and scaling patterns [9]. Dermoscopy has emerged as an invaluable extension of clinical examination, allowing real-time visualization of subsurface pigmentary, follicular, vascular, and scaling patterns that are not appreciable to the naked eye [2]. In addition to improving diagnostic confidence, dermoscopy serves as an effective patient education tool by enabling clinicians to demonstrate characteristic findings during consultation. This visual explanation often reassures patients, reduces anxiety, and facilitates counseling regarding prognosis and management [10].

Although histopathological examination remains the reference standard for selected disorders, skin biopsy is invasive and may not always be feasible or acceptable, particularly for lesions involving cosmetically sensitive sites such as the face [10,11]. Dermoscopy therefore serves as an effective non-invasive bridge between conventional clinical examination and an invasive biopsy by allowing real-time visualization of subsurface changes [2,12].

The dermoscopic findings in the present study were in concordance with those reported in previous literature, and no novel dermoscopic features were identified [3,4,6,13-18]. This highlights the consistency and reproducibility of characteristic dermoscopic patterns across different populations and supports their application in the routine evaluation of hypopigmentary and depigmentary disorders. For ease of clinical application, the key dermoscopic clues are summarized in Table 4 as a practical bedside reference.

**Table 4:** Summary of key dermoscopic features of common hypopigmentary and depigmentary disorders based on our study ± indicates a variable finding (may or may not be present).

Dermoscopic parameters	Vitiligo	Idiopathic guttate hypomelanosis	Pityriasis alba	Pityriasis versicolor	Contact leukoderma	Nevus depigmentosus	Leprosy
Background color	Diffuse white glow	Diffuse white glow or white	Brownish-white	Brownish-white	Diffuse white glow or white	Brownish-white	Brownish-white
Pigment network	Absent usually; reverse pigment network in a few	Absent	Faint	Faint	Absent	Preserved reticular network or faint network	Distorted
Margin characteristics	Variable (ill-defined to well-defined)	Well-defined margins with amoeboid, feathery, and petaloid patterns	Ill-defined	Ill-defined	Ill-defined	Feathery margins with pseudopod-like extensions	Ill-defined margins
Scaling	Absent	Absent	Diffuse, fine, white scales	Diffuse white scales; scaling in skin creases	Absent	Absent	Absent
Vessels	±, linear > dotted	Absent	May be present	May be present	±, linear > dotted	Absent	Linear vessels
Follicular findings	Leucotrichia ±; perifollicular pigmentation ±	Perifollicular pigmentation ±	No changes	Perifollicular scaling ±	No changes	Perifollicular pigmentation ±	Reduced appendages
Supportive dermoscopic clues	Diffuse white glow; reverse pigment network	Amoeboid/petaloid/feathery margins	Ill-defined margins with diffuse, fine scales	Scaling accentuated in skin creases; perifollicular scaling	Less homogeneous white glow	Pseudopod-like extensions	Yellowish-orange structureless areas

Our findings reinforce that diagnosis of hypopigmentary disorders relies on recognizing characteristic combinations of dermoscopic features interpreted alongside clinical examination, Wood's lamp evaluation, and KOH microscopy. However, the findings should be interpreted in light of certain limitations, including the relatively small sample size for some disease categories and the lack of histopathological confirmation in all cases. In addition, overlap of dermoscopic features among certain disorders may occasionally lead to diagnostic ambiguity and interobserver variability.

## Conclusions

Dermoscopy is a rapid and non-invasive modality that provides useful adjunctive information in the evaluation of hypopigmentary and depigmentary disorders. Characteristic dermoscopic patterns may aid in the differentiation of clinically overlapping disorders. Combined with Wood’s lamp examination and KOH microscopy, dermoscopy may serve as a useful adjunct in routine dermatological evaluation of pigmentary disorders. Further prospective multicentric studies involving larger cohorts and diverse skin phototypes, using appropriate reference standards, are warranted to establish its incremental diagnostic value and validate the reproducibility and generalizability of these findings.
